# Apparent diffusion coefficient measurement covering complete tumor area better predicts rectal cancer response to neoadjuvant chemoradiotherapy

**DOI:** 10.3325/cmj.2015.56.460

**Published:** 2015-10

**Authors:** Ivana Blažić, Ružica Maksimović, Milan Gajić, Đorđije Šaranović

**Affiliations:** 1 Clinical Hospital Centre Zemun, Belgrade, Serbia; 2 Clinical Centre of Serbia, Centre for Radiology and Magnetic Resonance Imaging, Belgrade, Serbia; 3 Institute for Medical Statistics and Informatics, Belgrade, Serbia

## Abstract

**Aim:**

To determine the impact of two apparent diffusion coefficient **(**ADC) measurement techniques on diffusion-weighted magnetic resonance images (DW MRI) on the assessment of rectal cancer response to neoadjuvant chemoradiotherapy (CRT).

**Methods:**

ADC values were measured prospectively with two different techniques – the first, which measures ADCs in the most cellular tumor parts, and the second, which measures the entire tumor area, in 58 patients with locally advanced rectal cancer on pre-CRT and post-CRT image sets. Areas under the receiver operating characteristic curves (AUCs) and parameters of diagnostic accuracy were calculated for pre- and post-CRT ADC values and numeric and percent ADC change for each technique to determine their performance in tumor response evaluation using histopathological tumor-regression grade as the reference standard.

**Results:**

The second technique yielded higher AUCs (0.935 vs 0.704, *P* < 0.001), percent-change (0.828 vs 0.636, *P* < 0.001), and numeric-change (0.866 vs 0.653, *P* < 0.001) than the first technique for post-CRT ADC. Accuracies for post-CRT ADC assessment were 62% for the first and 88% for the second technique (cut-off values: 0.98 and 1.29 × 10^−3^ mm^2^/s, respectively) and for ADC change assessment, both numeric and percent, 59% and 74%, respectively (cut-off values: increase of 0.18 and 0.28 × 10^−3^ mm^2^/s; increase of 24% and 37%, respectively).

**Conclusions:**

The type of measurement technique significantly affected ADC results. ADC measurements covering a larger area better predicted tumor response to therapy. Post-CRT ADCs, regardless of the measurement technique, and numeric ADC change measured in the whole tumor volume accurately identified non-complete responders. Post-CRT ADCs measured in the entire tumor area yielded the highest accuracy level in tumor response evaluation.

Neoadjuvant long course combined chemoradiotherapy (CRT), as a first step of a standard treatment for locally advanced rectal cancer, aims to downsize and downstage tumor before surgery in order to increase the number of complete resections with no microscopic residual tumors (R0 resections) and thereby reduce local recurrence rate. In locally advanced tumors, this treatment often leads to excellent response. In up to 24% of patients, no residual tumor tissue is found in the resection specimen, corresponding to complete tumor response (1). These patients have a better prognostic outcome than patients with residual tumor. Also, in patients with complete or significant response to CRT, organ saving treatment may be applied (1-6). To safely perform less invasive strategies, it is crucial to accurately select patients and reliably assess tumor response to treatment (7,8). Tumor response is estimated by different imaging techniques. Since conventional magnetic resonance imaging (MRI) is not sufficient for this purpose, functional MRI techniques, such as diffusion-weighted imaging (DWI) and dynamic-contrast enhanced MRI, have been used (9-12). DWI analyzes extracellular movement of water protons, which depends on tissue microarchitecture and can be quantitatively measured as apparent diffusion coefficient (ADC). It allows assessment of tumor response to treatment due to its superb tumor tissue definition as high signal intensity areas and quantitative information reflecting tissue cellularity. ADC has become an established oncologic biomarker, as lower ADC values indicate malignant tissues (13-16). ADC measurements are performed by positioning a region of interest (ROI) in the tumor area and measuring ADC values within the ROI. However, it is still debatable whether the measurements should be performed within the most cellular tumor parts represented as markedly restricted diffusion zones or in the larger tumor areas with inclusion of necrotic and fibrotic tumor parts. Therefore, the aim of our study is to determine the impact of two different ADC measurement techniques on DW MR images on the assessment of rectal cancer response to neoadjuvant CRT.

## Materials and methods

### Patient selection criteria

Between June 2012 and January 2015 eighty patients with locally advanced rectal cancer (LARC) diagnosed at Centre for Radiology and Magnetic Resonance Imaging, Clinical Centre of Serbia were prospectively evaluated. The study was approved by institutional review board and all patients signed written informed consent. Inclusion criteria were 1) rectal adenocarcinoma detected by digital rectal examination or endoscopy and histopathologically confirmed, 2) locally advanced disease staged at pre-CRT T2-weighted MRI as T3-T4 and/or positive nodal stage (one or more lymph nodes larger than 5 mm and/or exhibiting heterogeneous signal intensity or an irregular border), 3) neoadjuvant treatment consisting of a long course CRT (total radiation dose of 50.4 Gy in 28 fractions, daily dose of 1.8 Gy, with concomitant chemotherapy during the first and last weeks of radiation therapy [5-fluorouracil at the dose of 225 mg/m^2^/d, 5 days per week]), 4) surgical resection performed after completion of CRT. Twenty-two patients were excluded because of metastatic disease (12 patients), insufficient MR and/or DW image quality (5 patients), non-resectable tumor (3 patients), or mucinous subtype of rectal cancer exhibiting predominantly high T2w signal intensity with very high ADC values as a consequence of the very low cellular density (2 patients). The final study group consisted of 58 patients (38 male; mean age 61.3 years; standard deviation 11.8 years).

### Reference standard

Histopathological staging served as a standard of reference and was made according to the tumor regression grade (TRG) (17). Grading of response was as follows: TRG 1 (complete regression), rectal tissue specimens without viable tumor cells; TRG 2, single cells or small groups of cells within rectal tissue specimen; TRG 3, residual cancer outgrown by fibrosis; TRG 4, significant fibrosis outgrown by tumor tissue; and TRG 5, extensive residual cancer without fibrosis. Responder group consisted of TRG 1 and TRG 2 tumors, which could be eligible for organ saving treatment, while non-responder group consisted of TRG 3, TRG 4, and TRG 5 tumors. In the non-responder group, tumors were further subdivided into partial responder group (TRG 3) and poor responder group (TRG 4 and TRG 5).

### MRI technique

Before examination, all patients underwent bowel cleansing and received 20 mg of spasmolytic agent (hyoscine butilbromide, Buscopan, BoehringerIngelheim) intravenously shortly before MRI to avoid motion artifacts due to bowel peristalsis. MRI examinations were performed at 1.5 T (Magnetom, Avanto, Siemens Medical Systems, Erlangen, Germany) using a phased-array body coil and spine array coil to optimize signal-to-noise ratio. All patents underwent pre-treatment MRI for primary tumor and nodal staging and second MRI 7-9 weeks (median time 55 days, range 48-61 days) after completion of CRT for tumor restaging and evaluation of response to CRT. The median time between the first MR imaging and CRT was 23 days (range 18-41 days). The applied imaging protocol consisted of the following: T2-weighted turbo spin-echo (TSE) sequences in three orthogonal directions, high-resolution T2w TSE with small field of view set perpendicular to the tumor axis, and an axial DWI (single-shot echo planar imaging) sequence with b values of 50, 400, and 800 s/mm^2^, which was set and angulated identically to the high-resolution T2w TSE sequence. Parallel acquisition imaging (GRAPPA – generalized autocalibrating partially parallel acquisition) was applied to reduce the acquisition time and to improve image quality with acceleration factor PE = 2. ADC maps were generated automatically including all three diffusion sensitivity values in a monoexponential decay model. The sequence parameters are shown in Table 1.

**Table 1:** Magnetic resonance sequence parameters. Diffusion-weighted imaging was acquired with b values of 50, 400, and 800 s/mm^2^

Parameters	T2-weighted turbo spin echo	Diffusion-weighted imaging
Repetition time, ms	3000-3600	4400
Echo time, ms	89-93	76
Echo trains per slice, n	14	1
Matrix size	208x260/169x210	296x379
Field of view, mm	210-260	380
Receiver bandwidth, Hz/pixel	120	1628
Number of excitation	2	2
Slice thickness, mm	3-5	4
Distance factor, %	10	30
Number of slices	25-40	34
Echo planar imaging factor	150	-
Acquisition time, min	2.54-3.29	1.40

### Image analysis and ADC measurement techniques

**Figure 1:**
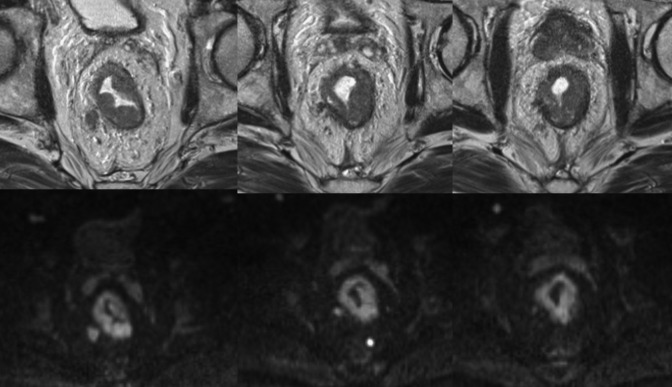
Areas of rectal cancer tissue on T2-weighted images (top row) corresponded to high signal intensity areas on diffusion-weighted images (bottom row) in a tumor estimated as T3a on pre-chemoradiotherapy magnetic resonance.

**Figure 2:**
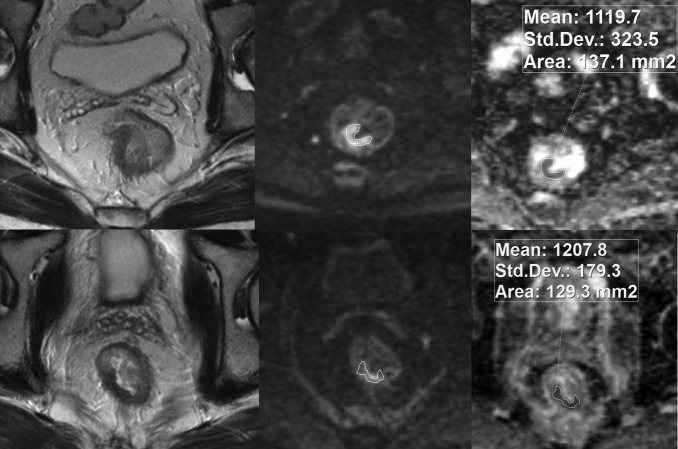
Pre-chemoradiotherapy (CRT) and post-CRT T2-weighted (left column), diffusion-weighted images (middle column), and apparent diffusion coefficient (ADC) (right column) image sets of rectal cancer estimated at pre-CRT magnetic resonance imaging as T3a from patient who experienced good response to CRT (TRG 2). Numbers listed in the ADC images indicate particular regions of interest.

All images in pre- and post-treatment sets were reviewed by one radiologist with six-year extensive experience in rectal cancer imaging, who was blinded to the patients’ clinical data and pathology reports. Tumor was identified on T2w images as a tissue exhibiting higher signal intensity than the muscular layer of the adjacent rectal wall, which corresponds to the high signal intensity area on DW images (Figure 1). ROIs were manually positioned on b 800 DW images and then copied and pasted to the corresponding ADC maps, due to the higher resolution of DW images. Post-CRT measurements were performed after having reviewed pre-CRT MRI in order to position the ROI within the location of the primary tumor (Figure 2). When high signal intensity zones were not identified on post-CRT DWI, ROIs were placed in the rectal wall at the former position of tumor.

**Figure 3:**
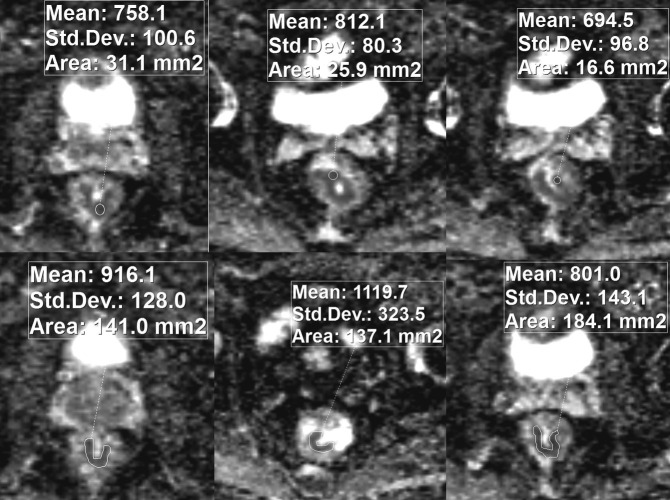
Demonstration of different apparent diffusion coefficient (ADC) measurement techniques obtained with circular (first technique) and freehand regions of interest (ROI) (second technique). Numbers listed in the ADC images indicate particular ROIs.

ADC measurements were performed on both pre-CRT and post-CRT image sets by two techniques. The first technique uses three circular ROIs positioned on three different slices containing tumor tissue within the areas of the most prominent restrictive diffusion (both on DW images and corresponding ADC maps, with exclusion of T2 shine-trough zones), which correspond to the most cellular tumor zones. Mean ADC value is calculated as average of three measured ADCs. The area of every single circular ROI was 10-50 mm^2^ (mean ROI area 27, standard deviation 7.23 mm^2^). The second technique uses a series of freehand ROIs defined on each slice containing tumor along the tumor borders in order to include the entire tumor area in the measurement (mean sum of ROI areas 3689 mm^2^, standard deviation 956 mm^2^) (Figure 3). Mean tumor ADC value is calculated as average of the measured ADC values in each section to obtain the representative ADC value of heterogeneous tumor tissue.

### Statistical analysis

All statistical analyses were performed using SPSS (version 20, IBM Corp., Armonk, NY, USA) and MedCalc statistical software (version 14.8.1, MedCalc Software, Ostend, Belgium). Paired samples *t* test was performed to compare the mean pre- and post-CRT ADC values obtained by each technique. Independent samples *t* test was used to compare the mean ADC values and numeric and percent values of ADC change obtained by each technique in responder and non-responder group. To evaluate diagnostic performance of each measurement technique in assessment of tumor response to therapy and to determine the optimal cut-off value in discriminating different levels of tumor response to therapy, receiver operating characteristic (ROC) curves were generated for pre-CRT and post-CRT measurements as well as for the numeric values and percentage of the ADC change. The area under the ROC curve (AUC) was considered as relative diagnostic accuracy. To compare relative diagnostic accuracy, pair-wise comparison of the ROC curves was performed, with 95% confidence intervals (CI). Parameters of diagnostic accuracy (sensitivity, specificity, accuracy, positive predictive value, and negative predictive value) were calculated using the optimal cut-off value, which was determined according to the nearest point to the upper left corner in the ROC curve diagram. Differences in diagnostic performances of two techniques were analyzed by comparing ROC curves according to the method described by DeLong et al (18). Normally distributed data are presented as mean ± standard deviation, whereas data with not-normal distribution are presented as median and range. *P*-values lower than 0.05 were considered significant.

## Results

### Patients and treatment characteristics and histopathological findings

All patients underwent surgical resection after completion of neoadjuvant treatment. The median time between the second MRI and surgery was 12 days (range 4-37 days). Low anterior resection was performed in 45 patients, abdominoperineal resection in 11 patients, and extended resection in 2 patients. There were 9 tumors in the proximal rectum, 32 in the middle rectum, and 17 in the distal rectum. The mean distance from anal verge was 6.5 cm (standard deviation, 2.6 cm) and the mean tumor length was 3.2 cm (standard deviation, 1.1 cm). Histopathological analysis confirmed complete tumor response (ypT0/TRG 1) to CRT in 10 patients, grade TRG 2 in 9 patients, and partial or poor tumor response in 39 patients (TRG 3 22, TRG 4 15 and TRG 5 2 specimens) (Table 2).

**Table 2:** The pre-treatment magnetic resonance-estimated tumor and nodal stages and postoperative histopathological stages of the studied population

	T0	T1	T2	T3	T4	Total
**Pre-treatment** magnetic resonance imaging**+ diffusion-weighted imaging -estimated tumor and nodal stages**						
N0			n = 0	n = 2	n = 0	n = 2
N1			n = 2	n = 15	n = 0	n = 17
N2			n = 3	n = 30	n = 6	n = 39
Total			n = 5	n = 47	n = 6	n = 58
**Postoperative histopathological stages**						
N0	n = 9	n = 3	n = 9	n = 17	n = 1	n = 39
N1	n = 1	n = 0	n = 2	n = 13	n = 1	n = 17
N2	n = 0	n = 0	n = 0	n = 2	n = 0	n = 2
Total	n = 10	n = 3	n = 11	n = 32	n = 2	n = 58

### Effects of different measurement techniques on ADC measurements

In the whole study group, there was no significant difference between percentage of ADC change measured with different techniques (*P* = 0.065). Second technique obtained significantly higher pre-CRT and post-CRT mean ADC values and numeric values of ADC change than first technique (*P* < 0.001) (Table 3).

**Table 3:** Mean apparent diffusion coefficient (ADC) values ( × 10^−3^ mm^2^/s ± standard deviation) obtained with different ADC measurement techniques (*P* values are for differences between responder and non- responder group)

Technique	All	Responders	Non-responders	*P*
**First**				
Pre-chemoradiotherapy ADC	0.79 ± 0.07	0.80 ± 0.08	0.78 ± 0.07	0.381
Post-chemoradiotherapy ADC	1.05 ± 0.15	1.13 ± 0.11	1.02 ± 0.15	**0.006**
Delta ADC	0.27 ± 0.15	0.33 ± 0.13	0.24 ± 0.15	**0.025**
Delta ADC (%)	34.6 ± 20.3	42.2 ± 18.6	31.0 ± 20.3	**0.048**
**Second**				
Pre-chemoradiotherapy ADC	0.87 ± 0.07	0.88 ± 0.07	0.87 ± 0.07	0.409
Post-chemoradiotherapy ADC	1.20 ± 0.17	1.36 ± 0.08	1.12 ± 0.14	**<0.001**
Delta ADC	0.33 ± 0.18	0.48 ± 0.10	0.26 ± 0.16	**<0.001**
Delta ADC (%)	38.4 ± 21.7	54.7 ± 13.8	30.4 ± 20.4	**<0.001**

### Quantitative analysis of tumor response to CRT

**Figure 4:**
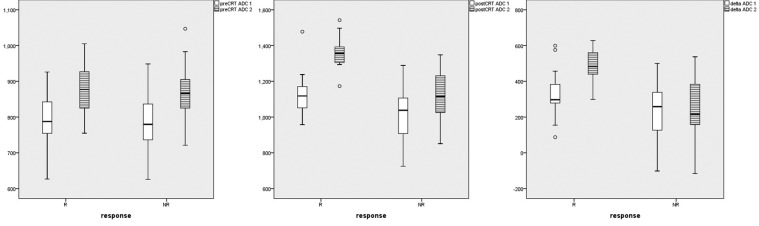
Boxplots comparing mean pre-chemoradiotherapy (CRT) apparent diffusion coefficient (ADC) values, mean post-CRT ADC values, and numeric values of ADC change between pre-CRT and post-CRT measurements obtained with two different ADC measurement techniques in the responder and non-responder group (ADC values in y-axis ×10^−6^ mm^2^/s).

There were no significant differences in mean (±standard deviation) pre-CRT ADC values between responder and non-responder group for both measurement techniques (for the first technique *P* = 0.381, for the second technique *P* = 0.409). There were also no significant differences between partial responder and poor responder subgroup of non-responder group for both measurement techniques (for the first technique 0.78 ± 0.07 vs 0.79 ± 0.08, for the second technique 0.86 ± 0.08 vs 0.88 ± 0.06; *P* = 0.760 and *P* = 0.311, respectively). Post-CRT ADC values were significantly higher in responder than in non-responder group (for the first technique *P* = 0.006, for the second technique *P* ≤ .001). Both numeric and percent ADC change was significantly higher in responder group for both measurement techniques (for the first technique *P* = 0.025 and 0.048, respectively, for the second technique both *P* < 0.001). Also, post-CRT measurements and numeric and percent ADC change were significantly higher in partial responder than in poor responder subgroup for both techniques (for the first technique *P* = 0.034, 0.024, and 0.034, respectively; for the second technique *P* = 0.002 for all measurements) (Figure 4).

**Figure 5:**
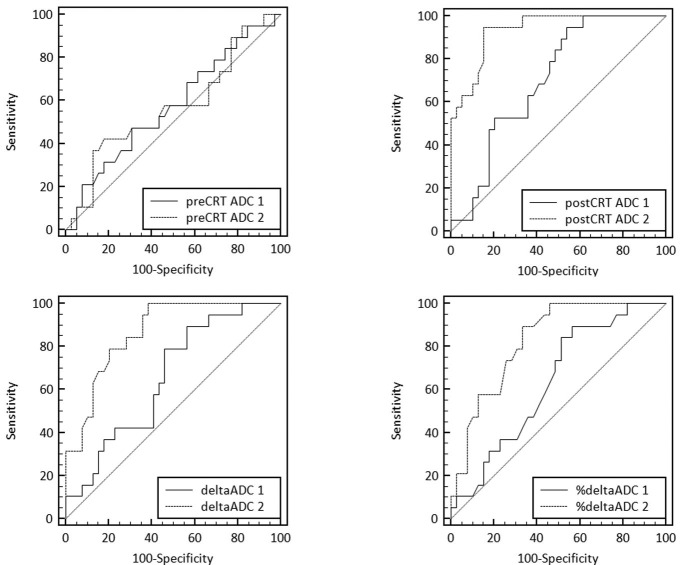
Comparison of receiver operating characteristic (ROC) curves demonstrating the diagnostic performance of each apparent diffusion coefficient (ADC) measurement technique in the assessment of tumor response to chemoradiotherapy (CRT) based on pre-CRT (area under the curve [AUC] 0.572 and 0.570, cut-off values 0.81 × 10^−3^ mm^2^/s and 0.91 × 10^−3^ mm^2^/s, respectively) and post-CRT ADC measurements (AUC 0.704 and 0.935, cut-off values 0.98 × 10^−3^ mm^2^/s and 1.29 × 10^−3^ mm^2^/s, respectively), numeric ADC change values (AUC 0.653 and 0.866, cut-off values +0.18 × 10^−3^ mm^2^/s and +0.28 × 10^−3^ mm^2^/s, respectively), and percent ADC change values (AUC 0.636 and 0.828, cut-off values +24% and +37%, respectively).

ROC curves were generated to evaluate diagnostic performance of ADC measurement techniques in the estimation of tumor response to CRT (Figure 5). Pre-CRT measurements resulted in AUC of 0.572 for the first technique and 0.570 for the second technique, which were not significantly different (*P* = 0.983). Post-CRT measurements resulted in AUCs of 0.704 for the first technique and of 0.935 for the second technique, showing significantly higher accuracy of the second technique (*P* < 0.001). This was further supported by reasonably high parameters of diagnostic accuracy (sensitivity 95%, specificity 85%, positive predictive value 75%, negative predictive value 97%, accuracy 88% for cut-off value of 1.29 × 10-3 mm^2^/s). The second technique demonstrated significantly higher diagnostic performance, with AUC of 0.866 and 0.828, for numeric and percent ADC change, respectively, than the first technique (AUC = 0.653 and AUC = 0.636, respectively; both *P* < 0.001). When a post-CRT ADC value of 1.29 × 10^−3^ mm^2^/s was used as a cut-off value for discriminating responder group from non-responder group, an accuracy of 88% (51/58) was obtained for the second technique. The same technique had an accuracy of 74% (43/58) for ADC change (both numeric and percent) for cut-off values of 0.28 × 10-3 mm^2^/s increase and 37% increase. The ADC measurements obtained by the first technique demonstrated lower diagnostic accuracy than those obtained by the second technique (for the pre- and post-treatment ADC measurements 62%, and for the measurements of ADC change 59%) (Table 4).

**Table 4:** Parameters of diagnostic performance of two measurement techniques for the assessment of tumor response to chemoradiotherapy (CRT) based on different apparent diffusion coefficient (ADC) measurements. Sensitivity, specificity, positive predictive value, negative predictive value, and accuracy were calculated using the optimal cut-off value listed for each item

	Area under the receiver operating characteristic curve (95% confidence intervals)	Sensitivity, % (n)	Specificity, % (n)	Positive predictive value, % (n)	Negative predictive value, % (n)	Accuracy, % (n)	Optimal cut-off value, mm^2^/s
**Pre-CRT ADC**							
1st technique	0.572 (0.435-0.701)	47 (9/19)	69 (27/39)	43 (9/21)	73 (27/37)	62 (36/58)	0.81 × 10^−3^
2nd technique	0.570 (0.433-0.699)	42 (8/19)	82 (32/39)	53 (8/15)	74 (32/43)	69 (40/58)	0.91 × 10^−3^
**Post-CRT ADC**							
1st technique	0.704 (0.570-0.817)	95 (18/19)	46 (18/39)	46 (18/39)	95 (18/19)	62 (36/58)	0.98 × 10^−3^
2nd technique	0.935 (0.837-0.983)	95 (18/19)	85 (33/39)	75 (18/24)	97 (33/34)	88 (51/58)	1.29 × 10^−3^
**Delta ADC**							
1st technique	0.653 (0.517-0.773)	90 (17/19)	44 (17/39)	44 (17/39)	90 (17/19)	59 (34/58)	+0.18 × 10^−3^
2nd technique	0.866 (0.715-0.941)	100 (19/19)	62 (24/39)	56 (19/34)	100 (24/0)	74 (43/58)	+0.28 × 10^−3^
**Delta ADC (%)**							
1st technique	0.636 (0.499-0.758)	90 (17/19)	44 (17/39)	44 (17/39)	90 (17/19)	59 (34/58)	+24%
2nd technique	0.828 (0.706-0.914)	90 (17/19)	67 (26/39)	57 (17/30)	93 (26/28)	74 (43/58)	+37%

## Discussion

To our knowledge, this is the first study comparing diagnostic performances of different ADC measurement techniques in the evaluation of tumor response to CRT. It demonstrated that two techniques yielded significantly different mean ADC values before and after CRT and numeric values of ADC change in the whole study group, indicating that ADC measurement technique considerably influences the measurement results. This finding is in concordance with the results of some previous studies, which showed that the variation in ROI size and positioning significantly influenced measurements (19,20). Our study demonstrated that the first ADC measurement technique, which measures the most viable solid tumor parts, was inferior in the assessment of rectal cancer response to neoadjuvant CRT to the second technique. The second technique had significantly higher accuracy in predicting the level of tumor response, which was highest in post-treatment measurements. These findings indicate that ADC measurements that cover the entire tumor volume together with necrotic and fibrotic regions provide better results in tumor response evaluation than measurements including only viable tumor areas. Similar results were obtained by Roth et al (21), who used an animal model to analyze pre-treatment and early post-treatment water diffusion parameters in response of colon carcinoma to different antineoplastic treatments. Also, Goh et al (19) demonstrated that ROI-outlined entire tumor was more reliable for CT perfusion measurements than the use of smaller ROIs.

Our study demonstrated that pre-treatment measurements did not reliably discriminate between responder group and non-responder group of tumors, regardless of the measurement technique. This was further confirmed by the finding that pre-CRT ADCs in subgroups of partial responders and poor responders in the non-responder group did not differ significantly for both measurement techniques. Regarding the accuracy of pre-CRT measurements, our results are concordant to the results from some previous studies that evaluated ADC measurements in the assessment of tumor response, although they did not assess different measurement techniques (22-24). On the other hand, Sun et al (25) reported significantly lower pre-treatment ADC values of tumors with good response to CRT. However, they considered tumor downstaging as a response to therapy; tumors were divided in T-downstaged group, consisting of tumors that lowered T status after CRT, and T non-downstaged group, consisting of tumors that showed the same or higher T status after CRT.

With regard to post-treatment measurements and measurements of ADC change, our study showed that the results of ADC measurements covering the entire tumor volume highly corresponded to the level of tumor response to therapy and were significantly more accurate than ADC measurements covering only solid tumor parts. Monguzzi et al (26) performed ADC measurements by placing three freehand ROIs outlying tumor on three slices containing the largest tumor area with diagnostic accuracy in tumor response evaluation of AUC = 0.82-0.83 and accuracy = 58%-74% for post-CRT measurements and AUC = 0.64-0.80 and accuracy = 81%-84% for measurements of ADC change, which corresponded to the results obtained by our second measurement technique (AUC = 0.935, accuracy = 88%; AUC = 0.828-0.866, accuracy = 74%, respectively). Ha et al (27) reported diagnostic performance of post-CRT ADC measurements obtained by placing at least four circular ROIs on each slice containing tumor (AUC = 0.705, accuracy = 67%), which is similar to our results for the first technique (AUC = 0.704, accuracy = 62%). Likewise, the recent results of Cai et al (28) showed AUC = 0.64-0.66 for post-CRT ADC measurements obtained by three small circular 4 mm^2^ ROI. Sun et al (25) demonstrated significant difference in the mean percentage of tumor ADC change between T downstaged and T non-downstaged group, indicating that the percentage of ADC increase during and after CRT may be a suitable marker of tumor downstaging. Our study used two measurement techniques and determined the accuracy for different levels of tumor response to therapy. Thus, it can be hypothesized that the larger measuring area yields more reliable results in terms of tumor tissue characterization after neoadjuvant CRT.

Our study obtained several clinically important results. We observed an excellent performance of post-CRT ADC measurements for selection of patients eligible for surgical treatment, demonstrated by very high negative predictive values (95 and 97% for the first and the second technique respectively), as well as an exquisite performance of numeric value of ADC change measured with the second technique, with negative predictive value of 100%. Also, we found no significant differences in diagnostic performance between post-treatment ADC measurements and measurements of ADC change obtained by the second technique, suggesting that only post-treatment ADC measurements, as a less time-consuming alternative, could be sufficient for tumor response evaluation, and that pre-treatment images do not necessarily have to be evaluated. This is supported by an appreciable level of all parameters of diagnostic accuracy of post-treatment ADC measured with the second technique. However, in complete or near-complete responders it could be very demanding to identify the former tumor position without comparison with pre-CRT images.

Our study has some limitations. Primarily, it was a single reader evaluation, so inter-observer variability was not an issue. ADC measurements are very subtle and subject to measuring errors, so inclusion of a second reader and evaluation of inter-observer variability and reproducibility of ADC measurements could improve the measurement objectivity. Also, the number of patients in every TRG group was relatively small and not sufficient to obtain an optimal threshold value for predicting tumor response to therapy. Thus, we formed a responder group with pathological complete response and almost complete response (TRG 1 and 2), even though it is debatable whether TRG 2 patients are eligible for less invasive treatment possibilities.

In conclusion, the novelty of this study is that two different tumor ADC measurement techniques were studied for the first time to estimate the level of rectal cancer response to neoadjuvant chemoradiotherapy. It was shown that the type of technique applied considerably influenced ADC measurements results and that measurement technique that covered larger area of the tumor better predicted tumor response to therapy. Post-treatment ADC measurements regardless of the applied technique and numeric value of ADC change measured in whole tumor volume can accurately identify non-complete responders eligible for surgical treatment. The highest accuracy in tumor response evaluation was obtained for post-treatment measurements when analyzing the entire tumor area.
